# Role of Microbial Enzymes in the Bioremediation of Pollutants: A Review

**DOI:** 10.4061/2011/805187

**Published:** 2011-09-07

**Authors:** Chandrakant S. Karigar, Shwetha S. Rao

**Affiliations:** Department of Biochemistry, Bangalore University, Bangalore 560001, India

## Abstract

A large number of enzymes from bacteria, fungi, and plants have been reported to be involved in the biodegradation of toxic organic pollutants. Bioremediation is a cost effective and nature friendly biotechnology that is powered by microbial enzymes. The research activity in this area would contribute towards developing advanced bioprocess technology to reduce the toxicity of the pollutants and also to obtain novel useful substances. The information on the mechanisms of bioremediation-related enzymes such as oxido-reductases and hydrolases have been extensively studied. This review attempts to provide descriptive information on the enzymes from various microorganisms involved in the biodegradation of wide range of pollutants, applications, and suggestions required to overcome the limitations of their efficient use.

## 1. Introduction

The quality of life on the Earth is linked inextricably to the overall quality of the environment. Unfortunately the progress in science, technology, and industry a large amount ranging from raw sewage to nuclear waste is let out or dumped into the ecosystem thereby posing a serious problem for survival of mankind itself on earth. 

In the past, wastes were traditionally disposed by digging a hole and filling it with waste material. This mode of waste disposal was difficult to sustain owing to lack of new place every time to dump. New technologies for waste disposal that use high-temperature incineration and chemical decomposition (e.g., base-catalyzed dechlorination, UV oxidation) have evolved. Although they can be very effective at reducing wide a range of contaminants but at the same time have several drawbacks. These methods are complex, uneconomical, and lack public acceptance. The associated deficiencies in these methods have focused efforts towards harnessing modern-day bioremediation process as a suitable alternative. 

Bioremediation is a microorganism mediated transformation or degradation of contaminants into nonhazardous or less-hazardous substances. The employability of various organisms like bacteria, fungi, algae, and plants for efficient bioremediation of pollutants has been reported [1, 2]. The involvement of plants in the bioremediation of pollutants is called as phytoremediation. The process of phytoremediation is an emerging green technology that facilitates the removal or degradation of the toxic chemicals in soils, sediments, groundwater, surface water, and air (RTDF). Genetically, engineered plants are also in use. For instance arsenic is phytoremediated by genetically modified plants such as *Arabidopsis thaliana* which expresses two bacterial genes. One of these genes allows the plant to modify arsenate into arsenite and the second one binds the modified arsenite and stores it in the vacuoles [2]. 

**Figure 1:**
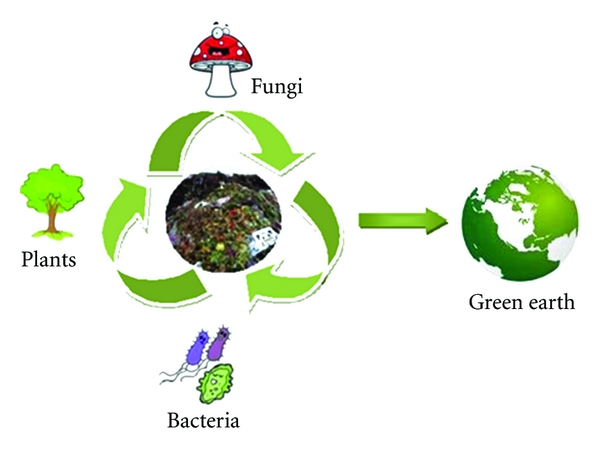
The process of waste bioremediation.

The process of bioremediation mainly depends on microorganisms which enzymatically attack the pollutants and convert them to innocuous products. As bioremediation can be effective only where environmental conditions permit microbial growth and activity, its application often involves the manipulation of environmental parameters to allow microbial growth and degradation to proceed at a faster rate (Figure 1).

The process of bioremediation is a very slow process. Only certain species of bacteria and fungi have proven their ability as potent pollutant degraders. Many strains are known to be effective as bioremediation agents but only under laboratory conditions. The limitation of bacterial growth is under the influence of pH, temperature, oxygen, soil structure, moisture and appropriate level of nutrients, poor bioavailability of contaminants, and presence of other toxic compounds. Although microorganisms can exist in extreme environment, most of them prefer optimal condition a situation that is difficult to achieve outside the laboratory [1, 3–5]. Most bioremediation systems operate under aerobic conditions, but anaerobic environments may also permit microbial degradation of recalcitrant molecules. Both bacteria and fungi rely on the participation of different intracellular and extracellular enzymes respectively for the remediation of recalcitrant and lignin and organopollutants [1, 6].

## 2. Enzymes

### 2.1. Introduction to Enzymes

Enzymes are biological catalysts that facilitate the conversion of substrates into products by providing favorable conditions that lower the activation energy of the reaction. An enzyme may be a protein or a glycoprotein and consists of at least one polypeptide moiety. The regions of the enzyme that are directly involved in the catalytic process are called the active sites. An enzyme may have one or more groups that are essential for catalytic activity associated with the active sites through either covalent or noncovalent bonds; the protein or glycoprotein moiety in such an enzyme is called the apoenzyme, while the nonprotein moiety is called the prosthetic group. The combination of the apoenzyme with the prosthetic group yields the holoenzyme.

### 2.2. Enzyme Nomenclature

Enzyme names apply to a single catalytic entity, rather than to a series of individually catalyzed reactions. Names are related to the function of the enzyme, in particular, to the type of reaction catalyzed [7].

### 2.3. Enzyme Classification

The ultimate identification of a particular enzyme is possible through its enzyme commission (E.C.) number. The assignment of E.C. numbers is described in guidelines set out by the International Union of Biochemistry. All known enzymes fall into one of these six categories. The six main divisions are (1) the oxidoreductases, (2) the transferases, (3) the hydrolases, (4) the lyases, (5) the isomerases, and (6) the ligases (synthetases). Oxidoreductases catalyze the transfer electrons and protons from a donor to an acceptor. Transferases catalyze the transfer of a functional group from a donor to an acceptor. Hydrolases facilitate the cleavage of C–C, C–O, C–N, and other bonds by water. Lyases catalyze the cleavage of these same bonds by elimination, leaving double bonds (or, in the reverse mode, catalyze the addition of groups across double bonds). Isomerases facilitate geometric or structural rearrangements or isomerizations. Finally, ligases catalyze the joining of two molecules [7]. 

## 3. Microbial Enzymes in Bioremediation

### 3.1. Microbial Oxidoreductases

The detoxification of toxic organic compounds by various bacteria and fungi [9] and higher plants [10] through oxidative coupling is mediated with oxidoreductases. Microbes extract energy via energy-yielding biochemical reactions mediated by these enzymes to cleave chemical bonds and to assist the transfer of electrons from a reduced organic substrate (donor) to another chemical compound (acceptor). During such oxidation-reduction reactions, the contaminants are finally oxidized to harmless compounds (ITRC 2002). 

The oxidoreductases participate in the humification of various phenolic substances that are produced from the decomposition of lignin in a soil environment. In the same way, oxidoreductases can also detoxify toxic xenobiotics, such as phenolic or anilinic compounds, through polymerization, copolymerization with other substrates, or binding to humic substances [11]. Microbial enzymes have been exploited in the decolorization and degradation of azo dyes [1, 12, 13].

Many bacteria reduce the radioactive metals from an oxidized soluble form to a reduced insoluble form. During the process of energy production, bacterium takes up electrons from organic compounds and use radioactive metal as the final electron acceptor. Some of bacterial species reduce the radioactive metals indirectly with the help of an intermediate electron donor. Finally precipitant can be seen as the result of redox reactions within the metal-reducing bacteria [2].

Chlorinated phenolic compounds are among the most abundant recalcitrant wastes found in the effluents generated by the paper and pulp industry. These compounds are produced upon the partial degradation of lignin during pulp bleaching process. Many fungal species are considered to be suitable for the removal of chlorinated phenolic compounds from the contaminated environments. The activity of fungi is mainly due to the action of extracellular oxidoreductase enzymes, like laccase, manganese peroxidase, and lignin peroxidase, which are released from fungal mycelium into their nearby environment. Being filamentous, fungi can reach the soil pollutants more effectively than bacteria [14]. 

Water polluted with phenolic compounds can be de-contaminated by plants with the help of enzymes exuded by their roots. The plant families of *Fabaceae, Gramineae, *and *Solanaceae* are found to release oxidoreductases which take part in the oxidative degradation of certain soil constituents. Phytoremediation of organic contaminants has been generally focused on three classes of compounds: chlorinated solvents, explosives, and petroleum hydrocarbons [15, 16].

#### 3.1.1. Microbial Oxygenases

Oxygenases belong to the oxidoreductase group of enzymes. They participate in oxidation of reduced substrates by transferring oxygen from molecular oxygen (O_2_) utilizing FAD/NADH/NADPH as a cosubstrate. Oxygenases are grouped into two categories; the monooxygenases and dioxygenases on the basis of number of oxygen atoms used for oxygenation. They play a key role in the metabolism of organic compounds by increasing their reactivity or water solubility or bringing about cleavage of the aromatic ring. Oxygenases have a broad substrate range and are active against a wide range of compounds, including the chlorinated aliphatics. Generally the introduction of O_2_ atoms into the organic molecule by oxygenase results in cleavage of the aromatic rings. Historically, the most studied enzymes in bioremediation are bacterial mono- or dioxygenases. A detailed study of the role of oxygenases in biodegradation process is available [18–20]. 

Halogenated organic compounds comprise the largest groups of environmental pollutants as a result of their widespread use as herbicides, insecticides, fungicides, hydraulic and heat transfer fluids, plasticizers, and intermediates for chemical synthesis. The degradation of these pollutants is achieved by specific oxygenases. Oxygenases also mediate dehalogenation reactions of halogenated methanes, ethanes, and ethylenes in association with multifunctional enzymes [19].

#### 3.1.2. Monooxygenases

Monooxygenases incorporate one atom of the oxygen molecule into the substrate. Monooxygenases are classified into two subclasses based on the presence cofactor: flavin-dependent monooxygenases and P_450_ monooxygenases. Flavin-dependent monooxygenases contain flavin as prosthetic group and require NADP or NADPH as coenzyme. P_450_ monooxygenases are heme-containing oxygenases that exist in both eukaryotic and prokaryrotic organisms. The monooxygenases comprise a versatile superfamily of enzymes that catalyzes oxidative reactions of substrates ranging from alkanes to complex endogenous molecules such as steroids and fatty acids. Monooxygenases act as biocatalysts in bioremediation process and synthetic chemistry due to their highly region-selectivity and stereoselectivity on wide range of substrates. Majority of mono-oxygenase studied previously are having cofactor, but there are certain monooxygenases which function independent of a cofactor. These enzymes require only molecular oxygen for their activities and utilize the substrate as reducing agent [8, 22].

**Figure 2:**
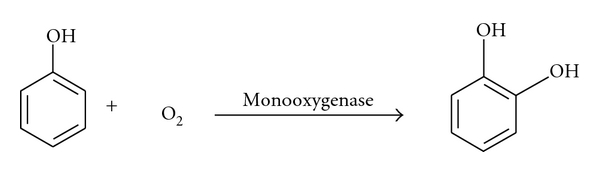
Degradation of aromatic compound by monooxygenase [8].

The desulfurization, dehalogenation, denitrification, ammonification, hydroxylation, biotransformation, and biodegradation of various aromatic and aliphatic compounds are catalyzed by monooxygenases. These properties have been explored in recent years for important application in biodegradation and biotransformation of aromatic compounds [8]. Methane mono-oxygenase enzyme is the best characterized one, among monooxygenases. This enzyme is involved in the degradation of hydrocarbon such as substituted methanes, alkanes, cycloalkanes, alkenes, haloalkenes, ethers, and aromatic and heterocyclic hydrocarbons [23, 24] (Figure 2). Under oxygen-rich conditions, mono-oxygenase catalyzes oxidative dehalogenation reactions, whereas under low oxygen levels, reductive dechlorination takes place. Oxidation of substrate can lead to de-halogenation as a result of the formation of labile products that undergo subsequent chemical decomposition [19, 25, 26].

#### 3.1.3. Microbial Dioxygenases

Dioxygenases are multicomponent enzyme systems that introduce molecular oxygen into their substrate. Aromatic hydrocarbon dioxygenases, belong to a large family of Rieske nonheme iron oxygenases. These dioxygenases catalyze enantiospecifically the oxygenation of wide range of substrates. Dioxygenases primarily oxidize aromatic compounds and, therefore, have applications in environmental remediation. All members of this family have one or two electron transport proteins preceding their oxygenase components. The crystal structure of naphthalene dioxygenase has confirmed the presence of a Rieske (2Fe–2S) cluster and mononuclear iron in each alpha subunit [4]. 

**Figure 3:**
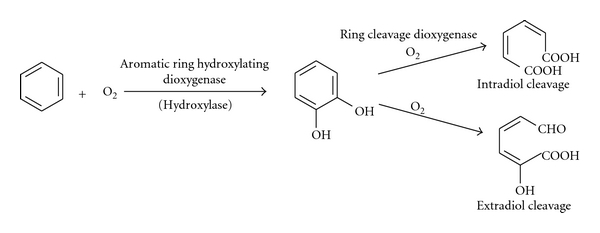
Degradation of aromatic compound by dioxygenase [17, 18].

The catechol dioxygenases serve as part of nature's strategy for degrading aromatic molecules in the Environment. They are found in the soil bacteria and involved in the transformation of aromatic precursors into aliphatic products. The intradiol cleaving enzymes utilize Fe(III), while the extradiol cleaving enzymes utilize Fe(II) and Mn(II) in a few cases [17] (Figure 3).

### 3.2. Microbial Laccases

Laccases (*p*-diphenol:dioxygen oxidoreductase) constitute a family of multicopper oxidases produced by certain plants, fungi, insects, and bacteria, that catalyze the oxidation of a wide range of reduced phenolic and aromatic substrates with concomitant reduction of molecular oxygen to water [9, 29]. Laccases are known to occur in multiple isoenzyme forms each of which is encoded by a separate gene [30], and in, some cases, the genes have been expressed differently depending upon the nature of the inducer [31].

**Figure 4:**
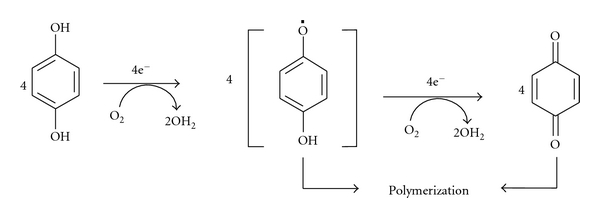
General reaction mechanism for phenol oxidation by laccase [21].

Many microorganisms produce intra and extracellular laccases capable of catalyzing the oxidation of ortho and paradiphenols, aminophenols, polyphenols, polyamines, lignins, and aryl diamines as well as some inorganic ions [29, 32, 33]. Laccases not only oxidize phenolic and methoxy-phenolic acids (Figure 4), but also decarboxylate them and attack their methoxy groups (demethylation). These enzymes are involved in the depolymerization of lignin, which results in a variety of phenols. In addition, these compounds are utilized as nutrients for microorganisms or repolymerized to humic materials by laccase [34]. Among the biological agents, laccases represent an interesting group of ubiquitous, oxidoreductase enzymes that show promise of offering great potential for biotechnological and bioremediation applications [9].

The substrate specificity and affinity of laccase can vary with changes in pH. Laccase can be inhibited by various reagents such as halides (excluding iodide), azide, cyanide, and hydroxide [35]. Different laccases appear to have differing tolerance toward inhibition by halides, indicating differential halide accessibility. Laccase production is sensitive to the nitrogen concentration in fungi. High nitrogen levels are usually required to obtain greater amounts of laccase. Recombinant laccase can be produced by either homologous or heterologous means [9].

### 3.3. Microbial Peroxidases

Peroxidases (donor: hydrogen peroxide oxidoreductases) are ubiquitous enzymes that catalyze the oxidation of lignin and other phenolic compounds at the expense of hydrogen peroxide (H_2_O_2_) in the presence of a mediator. These peroxidases can be haem and nonhaem proteins. In mammals, they are involved in biological processes such as immune system or hormone regulation. In plants, they are involved in auxin metabolism, lignin and suberin formation, cross-linking of cell wall components, defense against pathogens, or cell elongation [37, 38]. 

The hemeperoxidases have been classified into two distinct groups as found only in animals and found in plants, fungi, and prokaryotes. The second group peroxidases have been subdivided into three classes on the basis of sequence comparison. Class I is intracellular enzymes including yeast cytochrome *c* peroxidase, ascorbate peroxidase (APX) from plants, and bacterial gene-duplicated catalase peroxidases. Class II consists of the secretory fungal peroxidases such as lignin peroxidase (LiP) and manganese peroxidase (Mnp) from *Phanerochaete chrysosporium*, and *Coprinus cinereus* peroxidase or *Arthromyces ramosus* peroxidase (ARP). The main role of class II peroxidases appears to be the degradation of lignin in wood. Class III contains the secretory plant peroxidases such as those from horseradish (HRP), barley, or soybean. These peroxidases seem to be biosynthetic enzymes involved in processes such as plant cell wall formation and lignifications [37, 38].

Nonhaem peroxidases are not evolutionarily linked and form five independent families. They are thiol peroxidase, alkylhydroperoxidase, nonhaem haloperoxidase, manganese catalase and NADH peroxidase. Among all these thiol peroxidase is the largest and having two subfamilies such as glutathione peroxidases and peroxy redoxins [38].

#### 3.3.1. Classification of Peroxidase Enzymes

Peroxidases have been classified into many types based on its source and activity (http://peroxibase.toulouse.inra.fr/). Among peroxidases, lignin peroxidase (LiP), manganese-dependant peroxidase (MnP), and versatile peroxidase (VP) have been studied the most due to their high potential to degrade toxic substances in nature.

**(1) Microbial Lignin Peroxidases**

**Figure 5:**
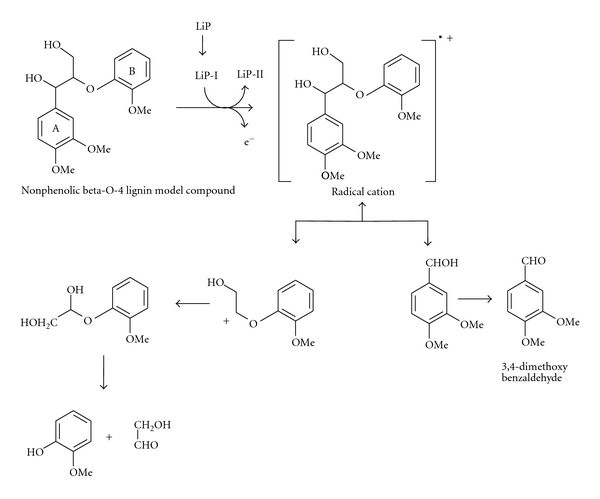
Lignin peroxidase (LiP)-catalyzed oxidation of nonphenolic *β*-O-4 lignin model compound [27].

Lignin peroxidases are heme proteins secreted mainly by the white rot fungus during secondary metabolism. In the presence of cosubstrate H_2_O_2_ and mediator like veratryl alcohol LiP degrade lignin and other phenolic compounds. During the reaction, H_2_O_2_ gets reduced to H_2_O with the gaining of electron from LiP, (which itself gets oxidized). The LiP (oxidized) with gaining an electron from veratryl alcohol returns to its native reduced state, and veratryl aldehyde is formed. Veratryl aldehyde then again gets reduced back to veratryl alcohol by gaining an electron from substrate. This result in the oxidation of halogenated phenolic compounds, polycyclic aromatic compounds and other aromatic compounds followed by a series of nonenzymatic reactions (Figure 5) [40, 41]. Lignin peroxidase (LiP) plays a central role in the biodegradation of the plant cell wall constituent lignin. LiP is able to oxidize aromatic compounds with redox potentials higher than 1.4 V (NHE) by single-electron abstraction, but the exact redox mechanism is still poorly understood [42].

**(2) Microbial Manganese Peroxidases**

**Figure 6:**
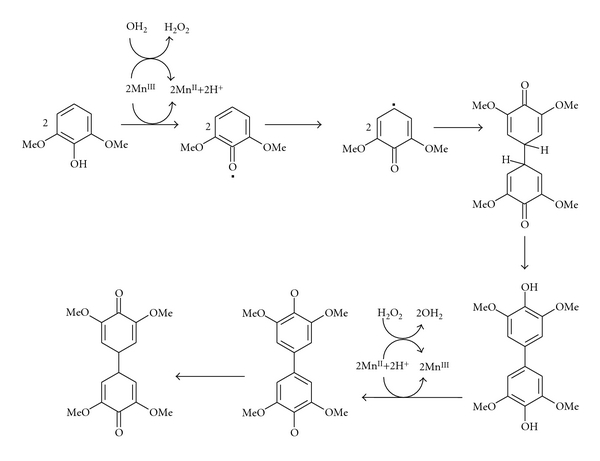
Proposed mechanism for the oxidation of 2,6-dimethoxyphenol by the MnP system [28].

MnP is an extracellular heme enzyme from the lignin-degrading basidiomycetes fungus, that oxidizes Mn^2+^ to the oxidant Mn^3+^ in a multistep reaction. Mn^2+^ stimulates the MnP production and functions as a substrate for MnP. The Mn^3+^, generated by MnP, acts as a mediator for the oxidation of various phenolic compounds. The resulting Mn³^+^ chelate oxalate is small enough to diffuse into areas inaccessible even to the enzyme, as in the case of lignin or analogous structures such as xenobiotic pollutants (Figure 6) buried deep within the soil, which are not necessarily available to the enzymes [41].

**(3) Microbial Versatile Peroxidases**

VP enzymes are able to directly oxidize Mn^2+^, methoxybenzenes, phenolic aromatic substrates like that of MnP, LiP, and horseradish peroxidase. VP has extraordinary broad substrate specificity and tendency to oxidize the substrates in the absence of manganese when compared to other peroxidases. It has also been demonstrated that VP is able to oxidize both phenolic and nonphenolic lignin model dimers [43]. Therefore, a highly efficient VP overproduction system is desired for biotechnological applications in industrial processes and bioremediation of recalcitrant pollutants [27, 44].

## 4. Microbial Hydrolytic Enzymes

The pollution of soil and water by industrial chemicals and petroleum hydrocarbons is a serious problem of the modern world. Due to their extensive use, they are found as environmental contaminants in numerous aquatic and terrestrial ecosystems. The use of bioremediation technologies for removing these contaminants provides a safe and economic alternative to commonly used physical-chemical treatment. Bacterial activity is the major process involved in the hydrolysis of organic pollutants (Table 1). Extracellular enzyme activity is a key step in degradation and utilization of organic polymers, since only compounds with molecular mass lower than 600 daltons can pass through cell pores [46].

Hydrolytic enzymes disrupt major chemical bonds in the toxic molecules and results in the reduction of their toxicity. This mechanism is effective for the biodegradation of oil spill and organophosphate and carbamate insecticides. Organochlorine insecticides such as DDT and heptachlor are stable in well-aerated soil but readily degrade in anaerobic environments [12, 46, 47]. Hydrolases also catalyze several related reactions including condensations and alcoholysis. The main advantages of this enzyme class are ready availability, lack of cofactor stereoselectivity, and tolerate the addition of water-miscible solvents. Hydrolases belong to group 3 of enzyme class and may further be classified according to the type of bond hydrolyzed [48]. 

Extracellular hydrolytic enzymes such as amylases, proteases, lipases, DNases, pullulanases, and xylanases have quite diverse potential usages in different areas such as food industry, feed additive, biomedical sciences, and chemical industries [49]. The hemicellulase, cellulase, and glycosidase are of much importance due to its application in biomass degradation [39].

### 4.1. Microbial Lipases

Lipase degrades lipids derived from a large variety of microorganisms, animals and plants. Recent works have shown that lipase is closely related with the organic pollutants present in the soil. Lipase activity was responsible for the drastic reduction total hydrocarbon from contaminated soil. Research undertaken in this area is likely to progress the knowledge in the bioremediation of oils spill [50, 51]. Lipases have been extracted from bacteria, plant, actinomycetes, and animal cell. Among these microbial lipases are more versatile because of their potent application in industries. These enzymes can catalyze various reactions such as hydrolysis, interesterification, esterification, alcoholysis and aminolysis [52].

Lipases are ubiquitous enzymes which catalyze the hydrolysis of triacylglycerols to glycerol and free-fatty acids. Lipolytic reactions occur at the lipid-water interface, where lipolytic substrates usually form equilibrium between monomeric, micellar, and emulsified states. Lipases have been classified into two types on the basis of criteria such as (a) enhancement in enzyme activity as soon as the triglycerides form an emulsion and (b) lipases with a loop of protein (lid) covering on the active site [53].

**Figure 7:**
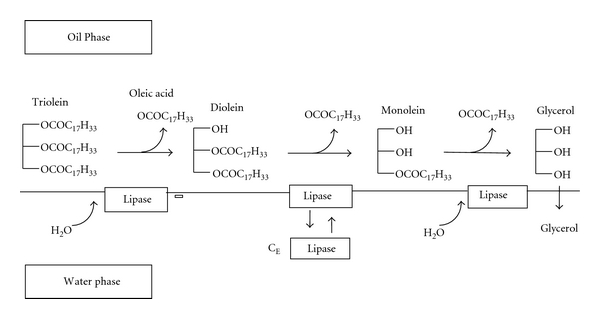
Proposed mechanism for triolein hydrolysis by *Candida rugosa* lipase in biphasic oil-water system. C_E_ represents the enzyme concentration in the bulk of the water phase [36].

 Triglyceride is the main component of natural oil or fat. This can hydrolyze consecutively to diacylglycerol, monoacylglycerol, glycerol, and fatty acids. Glycerol and fatty acids are widely used as raw materials, for instance, monoacylglycerol is used as an emulsifying agent in the food, cosmetic, and pharmaceutical industries. The study made on triolein hydrolysis from *Candida rugosa* lipase in the biphasic oil-water system as proven to be effective. The lipase adsorbs on to the oil-water interface in the bulk of the water phase. The lipase then breaks the ester bonds of triolein to produce consecutively diolein, monoolein, and glycerol. During the catalysis oleic acid is formed at each consecutive reaction stage. The glycerol formed is hydrophilic and thus dissolves into the water phase [36] (Figure 7).

Lipase activity was found to be the most useful indicator parameter for testing hydrocarbon degradation in soil [50, 51]. Lipase is of much interest in the production of regiospecific compounds which are employed in pharmaceutical industry. Along with its diagnostic usage in bioremediation, lipase has many potential applications in food, chemical, detergent manufacturing, cosmetic, and paper making industries, but its production cost has restricted its industrial use [53, 54]. 

### 4.2. Microbial Cellulases

Cellulases now promise the potential of converting waste cellulosic material into foods to meet burgeoning population and have been the subject of intense research [55]. Some organisms produce cell bound, cell envelope associated, and some extra cellular cellulases. Extracellular cellulases, hemicellulases, and pectinases have been shown to be constitutively expressed at very low levels by some bacteria and fungi [56, 57].

**Figure 8:**
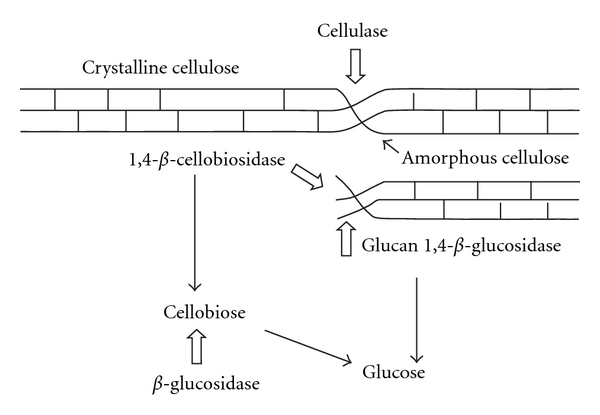
Proposed mechanism for the hydrolysis of cellulose by the fungal cellulase enzyme system [39].

Cellulases are usually a mixture of several enzymes. At least: three major groups of cellulases are involved in the hydrolysis process (1) endoglucanase (EG, endo- 1,4-D-glucanohydrolase) which attacks regions of low crystallinity in the cellulose fiber, creating free chain ends; (2) exoglucanase or cellobiohydrolase (CBH, 1,4-b-D-glucan cellobiohydrolase) which degrade the cellulose molecule further by removing cellobiose units from the free chain ends; (3) *β*-glucosidase which hydrolyzes cellobiose to glucose units. Along with major enzymes, some ancillary enzymes are also present. During the enzymatic hydrolysis, cellulose is degraded by the cellulases to reducing sugars (Figure 8) that can be fermented by yeasts or bacteria to ethanol [58].

Cellulase enzymes are capable of degrading crystalline cellulose to glucose. Cellulases have been used in the manufacture of detergents since early 1990s. Cellulases remove cellulose microfibrils, which are formed during washing and the use of cotton-based cloths. This is also observed as the colour brightening and material softening in the textile industry. Alkaline cellulases are produced by *Bacillus *strains and neutral and acidic cellulases by *Trichoderma *and *Humicola* fungi. In paper and pulp industry, cellulases have been employed for the removal of ink during recycling of paper. The cellulases are added during brewing to increase the juice liberation from fruit pulp and for the production of ethanol from cellulosic biomass [59].

### 4.3. Microbial Proteases

Proteases hydrolyze the breakdown of proteinaceous substance which enter atmosphere due to shedding and moulting of appendages, death of animals, and also as byproduct of some industries like poultry, fishery, and leather. Proteases belong to group of enzymes that hydrolyze peptide bonds in aqueous environment and synthesize them in nonaqueous environment. Proteases have wide range of applications in food, leather, detergent, and pharmaceutical industry [60, 61]. 

**Figure 9:**
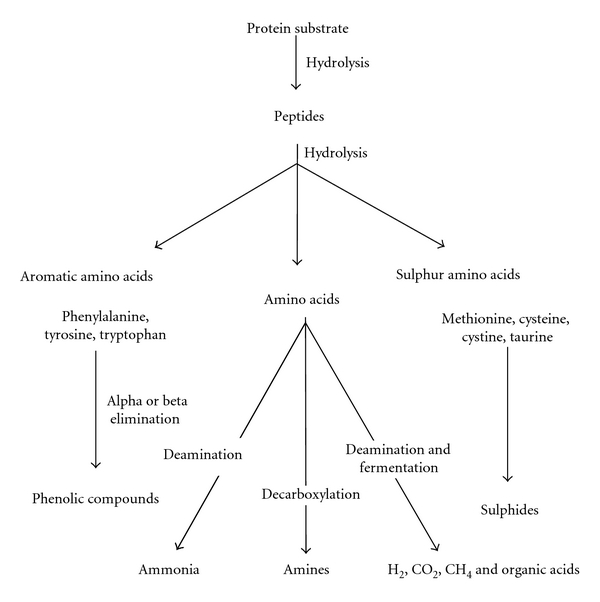
Proposed pathway for protease hydrolysis [45].

Proteases are divided as endopeptidases and exopeptidases based on the catalysis of peptide chain. Endo peptidases further grouped based on the position of active site such as serine endopeptidase, cysteine peptidase, aspartic endopeptidases, and metallopeptidases. The enzymes whose reaction mechanism is completely elucidated are grouped under (Figure 9). The exopeptidases act only near the terminal amino or carboxylic position of chain. The protease that acts on free amino, and carboxyl terminals are called as aminopeptidase and carboxypeptidase, respectively.

The endopeptidase acts on the inner regions of peptide chain. The presence of free amino and carboxyl terminal will have negative impact on enzyme activity [62]. 

Proteases have been used in the manufacture of cheese and detergent manufacturing industry since many years. The alkaline proteases are used in leather industry for the removal of hairs and parts which are present on the animal skin. Proteases have been employed for the production of dipeptide aspartame, which is a noncalorific artificial sweetener. In the pharmaceutical industry, a varying and specific proteases are used in developing effective therapeutic agents. Clostridial collagenase or subtilisin is used in combination with broad-spectrum antibiotics in the treatment of burns and wounds [63].

## Figures and Tables

**Table 1:** Industrial applications of microbial enzymes.

SI No.	Enzyme	Substrate	Reaction	Applications
1	Oxidoreductase			

1.1	Oxygenase			

1.1.1	Monooxygenase	Alkane, steroids, fatty acid, and aromatic compounds	Incorporation of oxygen atom to substrate and utilize substrate as reducing agent. Desulfurization, dehalogenation, denitrification, ammonification, and hydroxylation of substrate	Protein engineering, bioremediation, synthetic chemistry, and so forth.

1.1.2	Dioxygenase	Aromatic compounds	Introduction of two oxygen atom to the substrate results in intradiol cleaving and extradiol cleaving with the formation of aliphatic product	Synthetic chemistry, pharmaceutical industry, bioremediation, and so forth.

1.2	Laccase	Ortho and paradiphenols, aminophenols, polyphenols, polyamines, lignins, and aryldiamines	Oxidation, decarboxylation and demethylation of substrate.	Food industry, paper and pulp industry, textile industry, nanotechnology, synthetic chemistry, bioremediation, cosmetics, and so forth.

1.3	Peroxidase			

1.3.1	Lignin peroxidase	Halogenated phenolic compounds, polycyclic aromatic compounds and other aromatic compounds	Oxidation of substrate in the presence of cosubstrate H_2_O_2_ and mediator like veratryl alcohol.	Food industry, paper and pulp industry, textile industry, pharmaceutical industry, bioremediation, and so forth.

1.3.2	Manganese peroxidase	Lignin and other phenolic compounds	In the presence of Mn²^+^ and H_2_O_2_ the co-substrate catalyses oxidation of Mn²^+^ to Mn³^+^ which results in an Mn³^+^ chelateoxalate, which in turn oxidizes the phenolic substrates.	Food industry, Paper and pulp industry, textile industry, pharmaceutical industry, bioremediation, and so forth.

1.3.3	Versatile peroxidase	Methoxybenzenes and phenolic aromatic	The enzyme catalyzes the electron transfer from an oxidizable substrate, with the formation and reduction of compound I and compound II intermediates.	Industrial biocatalyst, bioremediation, and so forth.

2	Hydrolase			

2.1	Lipase	Organic pollutants such as oil spill	The hydrolysis of triacylglycerols to glycerols and free-fatty acids	Control of oil spills, detergent production, baking industry, paper and pulp industry, personal care products, and so forth.

2.2	Cellulase	Cellulosic substance	Hydrolyses the substrate to simple carbohydrates.	Textile manufacturing. detergent production, paper and pulp industry, bioremediation, and so forth.

2.3	Protease	Proteins	Enzymes that hydrolyze peptide bonds in aqueous environment.	Leather, laundry, biocatalyst, bioremediation, and so forth.
